# Characterization of calcineurin A and B genes in the abalone, *Haliotis diversicolor*, and their immune response role during bacterial infection

**DOI:** 10.7717/peerj.8868

**Published:** 2020-04-09

**Authors:** Tiranan Buddawong, Somluk Asuvapongpatana, Saengchan Senapin, Carmel McDougall, Wattana Weerachatyanukul

**Affiliations:** 1 Department of Anatomy, Faculty of Science, Mahidol University, Ratchathewi, Bangkok, Thailand; 2 Center of Excellence for Shrimp Molecular Biology and Biotechnology (Centex Shrimp), Faculty of Science, Mahidol University, Ratchathewi, Bangkok, Thailand; 3 National Center for Genetic Engineering and Biotechnology (BIOTEC), National Science and Technology Development Agency (NSTDA), Klongluang, Pathumthani, Thailand; 4 Australian Rivers Institute, Griffith University, Nathan, Queensland, Australia

**Keywords:** Calcineurin, CNA, CNB, Immune response, *Vibrio parahaemolyticus*

## Abstract

Calcineurin (CN) is known to be involved in many biological processes, particularly, the immune response mechanism in many invertebrates. In this study, we characterized both *HcCNA* and *HcCNB* genes in *Haliotis diversicolor*, documented their expression in many tissues, and discerned their function as immune responsive genes against *Vibrio parahaemolyticus* infection. Similar to other mollusk CNs, the *HcCNA* gene lacked a proline-rich domain and comprised only one isoform of its catalytic unit, in contrast to CNs found in mammals. HcCNB was highly conserved in both sequence and domain architecture. Quantitative PCR and in situ hybridization revealed that the genes were broadly expressed and were not restricted to tissues traditionally associated with immune function. Upon infection of *H. diversicolor* with *V. parahaemolyticus* (a bacteria that causes serious disease in crustaceans and mollusks), both *HcCNA* and *HcCNB* genes were highly up-regulated at the early phase of bacterial infection. *HcCNB* was expressed significantly higher than *HcCNA* in response to bacterial challenge, suggesting its independent or more rapid response to bacterial infection. Together, the two *CN* genes are unique in their gene structure (particular *HcCNA*) and distribution in mollusk species and likely function as immune responsive genes along with many other genes that are enhanced in the early phase of *V. parahaemolyticus* infection in abalone.

## Introduction

Calcineurin (CN) is a calcium/calmodulin-dependent serine/threonine protein phosphatase comprising two subunits (A and B), both of which are highly conserved from yeast to humans (Klee, Crouch & Krinks, 1979; Klee, Ren & Wang, 1998). CNA (57–71 kDa) contains phosphatase, CNB-binding, CaM-binding, and autoinhibitory domains, while the smaller CNB (16–19 kDa) consists of four EF-hand type calcium-binding motifs (Rusnak & Mertz, 2000). In vertebrates, CN plays roles in regulating several cellular activities through dephosphorylation of target proteins in a calcium- and calmodulin-dependent manner (Feske et al., 2003). Within the immune system, CN acts by regulation of the ‘nuclear factor of activated T cells’ (NFAT) transcription factor family (Rao, Luo & Hogan, 1997). Upon dephosphorylation by CN, the activated NFAT protein translocates into the nucleus to regulate target gene expression (Feske et al., 2003). CN has also been found to function in neuronal metabolism, cell cycle control, vesicular trafficking, muscle hypertrophy, bone formation and absorption, and T cell activation (Zhang et al., 1996; Kayyali et al., 1997; Sugiura et al., 2001; Shibasaki, Hallin & Uchino, 2002; Kuroda et al., 2008; Yamanaka et al., 2008). Additionally, its function in innate immunity in neutrophils and in T-lymphocyte activation has been established (Clipstone & Crabtree, 1992; Greenblatt et al., 2010).

CN subunits have been identified in invertebrates. In *Drosophila melanogaster* and *Ceanorhabditis elegans* CN has been implicated in a range of functions including muscle formation and function, nervous system function, and germline development (Bandyopadhyay et al., 2002; Kuhara et al., 2002; Gajewski et al., 2003). In crabs, bacterial challenge experiments resulted in the up-regulation of CNA and CNB in immune related tissues such as haemocytes, gill and hepatopancreas (Li et al., 2015), indicating that invertebrate CN may function in immunity. In mollusks, CNA and CNB subunits have been identified in the pearl oyster, *Pinctada fucata*, and expression of CNA was detected in a number of tissues including the mantle, gills, adductor muscle, foot, digestive gland, and gonad (Li et al., 2009). Inhibition of CN in this species revealed that it plays pivotal roles in both shell formation and the mediation of the immune response of hemocytes (Li et al., 2010a; Li et al., 2010b).

In this study, we asked whether both CNA and CNB are expressed in immune-related tissues of the gastropod, *Haliotis diversicolor*, and whether they are involved in an immune response of these animals. The immune response of this species is of interest because *H. diversicolor* is an economically important aquaculture species in the South of Thailand. Prevention of microbial infection is a primary concern. It has been reported in Japan and Taiwan that *V. parahaemolyticus* can infect abalone, causing ulcers in mantle tissue, white spots on the foot, and withering syndrome (Nishimori et al., 1998; Liu et al., 2000; Huang, Liu & Lee, 2001). In addition, infection of *V. parahaemolyticus* posts a significant risk for abalone aquaculture (Cai, Han & Wang, 2006). Although *V. parahaemolyticus* is a pathogen normally associated with shrimp farming, it is frequently found in the stools of humans suffering gastroenteritis after ingestion of undercooked fish and shellfish (Chiou, Chen & Chen, 1991). Here, we mimicked *V. parahaemolyticus* infection in the abalone and investigated whether CNA and CNB might play a role in the immune response during this bacterial infection. Enhancement of CNA and CNB genes in conjunction with other immune-related genes should be a future developing strategy to fight against this severe bacterial infection in abalone aquaculture.

## Material and Methods

### Experimental animals, RNA extraction, and cDNA synthesis

The experimental procedure was approved by the Faculty of Science, Mahidol University Animal Care and Use Committee (SCMU-ACUC, Protocol Number MUSC60-040-390). Adult healthy *H. diversicolor* (55.0  ± 5.0 mm in shell length and 10.0  ± 3.0 gm in wet weight) were reared at Phuket Abalone Farm, Phuket, Thailand. They were maintained in seawater in polyethylene tanks at 23−25 °C with a salinity of 28–30 ppt and fed daily with fresh kelp before the experiments. The hemolymph was collected from the pericardial cavities into anticoagulant (383 mM NaCl, 115 mM glucose, 37 mM C_6_H_7_NaO_7_, 11 mM EDTA) and centrifuged immediately (800 × g, 10 min, 4 °C) to collect the hemocytes. The mantle, gill, epipodium, hepatopancreas, gonad, stomach, foot, kidney, and hypobranchial gland were carefully dissected and stored in RNAlater RNA stabilization reagent (Ambion, Austin, TX) for further RNA extraction. Total RNA was extracted with Trizol reagent (Invitrogen, Carlsbad, CA) according to the manufacturer’s protocol and treated with DNAse I (Thermo Fisher Scientific, Carlsbad, CA) to remove genomic DNA. RNA was reverse-transcribed into cDNA using the SuperScript III First-Strand Synthesis System for RT-PCR (Invitrogen, Carlsbad, CA) following the manufacturer’s instructions.

### Molecular cloning of *H. diversicolor* CNA (*HcCNA*) and CNB (*HcCNB*)

Partial sequences of *HcCNA* and *HcCNB* were obtained by RT-PCR using pairs of primers specified in Table 1. Primers were designed based on available nucleotide sequences of CNA and CNB in *Haliotis discus discus* (GenBank accession numbers EF103366 and EF103365, respectively). A 50 µl reaction solution of SuperScript™ III One-Step RT-PCR System with Platinum™ Taq DNA Polymerase (Invitrogen, Carlsbad, CA) contained 1 µg of the mixed mantle RNA isolated from 3 adult healthy *H. diversicolor*, 0.2 µM of each primer, 2 µl of SuperScript III RT/Platinum Taq mix, 25 µl of 2 × reaction mix, and autoclaved distilled water. The PCR reaction was carried out under the following conditions: 50 °C for 30 min, 94 °C for 2 min, 40 cycles of 94 °C for 15 s, 55 °C for 30 s, and 68 °C for 1 min and final extension at 68 °C for 5 min. The PCR products were electrophoresed in a 1.2% agarose gel and the band of 1,600 bp (for CNA) or 1,500 bp (for CNB) was excised and purified using a FavorPrep GEL/PCR Purification Kit (Favorgen, Ping-Tung, Taiwan). The purified PCR products were subsequently cloned into pDrive Cloning Vector (Qiagen, Chatsworth, CA) and were Sanger sequenced by 1st BASE Company (Seri Kembangan, Selangor, Malaysia).

**Table 1:** Oligo nucleotide primers used in this study.

Primer name	Nucleotide sequence (5′ → 3′)	Purpose
CNA-F	GAAGGCACTCACACCTATTGC	Cloning
CNA-R	CAGTTTGAAATGTGTACAGCCATA	
CNB-F	GGGGGTCTTCGATCTATTAATATGGG	Cloning
CNB-R	TGTATCCACTACCCACCAACAC	
*HcCNA*-F	AGGTGATCCGCAACAAAATC	Real-time PCR/*In situ* hybridization
*HcCNA*-R	TCCTCCAGACAACACACCAA	
*HcCNB*-F	CAGTTTGCCAATGGAGCTTT	Real-time PCR/*In situ* hybridization
*HcCNB*-R	CTCTCTGCACCAGTGGGTTT	
*β-actin*-F	ACCACGGGTATTGTTCTTGAC	Reference gene
*β-actin*-R	CGGTGGTGGTGAAGGAGTAAC	

### Sequence alignment and phylogenetic analysis

Translation to amino acid sequence was carried out using the translation tool at http://web.expasy.org/translate/, and prediction of protein domains was carried out using the SMART tool (http://smart.embl-heidelberg.de/). Molecular weight and isoelectric point prediction were carried out at http://web.expasy.org/protparaml. Protein sequence similarity searches were performed by using BLAST software (http://blast.ncbi.nlm.nih.gov/Blast.cgi), and multiple sequence alignments were generated using Clustal Omega (http://www.ebi.ac.uk/Tools/msa/clustalo/). Maximum likelihood phylogenetic analysis was conducted using Molecular Evolutionary Genetics Analysis (MEGA 7) software (Kumar, Stecher & Tamura, 2016) using 1000 bootstrap replicates based on JTT matrix-based model (Jones, Taylor & Thornton, 1992) and Le-Gascuel-2008 model (Le & Gascuel, 2008) for HcCNA and HcCNB, respectively.

### *HcCNA* and *HcCNB* mRNA expression analysis by quantitative real-time PCR

Real-time PCR analysis was used to quantify the expression level of *HcCNA* and *HcCNB* in cDNA from several tissues of 3 healthy adult *H. diversicolor* using pairs of specific primers for *HcCNA*-F, *HcCNA*-R and *HcCNB*-F, *HcCNB*-R (Table 1). Real-time PCR was performed in triplicate using Luna Universal qPCR Master Mix (New England Biolabs, Ipswich, MA). The 20 µl reaction mixture contained 0.5 µl of cDNA, 10 µl of Luna Universal qPCR mix, 0.5 µl of each primer, and 8.5 µl of PCR grade water. The real-time PCR cycles were 95 °C for 1 min, 45 cycles of 95 °C for 15 s, 60 °C for 30 s, and 72 °C for 30 s using a Bio-Rad CFX96 Touch Real-Time PCR Detection System (Bio-Rad Laboratories, Inc., Hercules, CA). In order to assess the specificity of PCR amplification, a melting curve analysis was performed at a final single cycle by increasing the temperature from 60 °C to 95 °C in the rate of 0.05 °C/s. The baseline was set automatically by Bio-Rad CFX manager software (version 3.1). Relative expression in different tissues was calculated by a Livak (2^−ΔΔ*Cq*^) method using the quantification cycle (Cq) values of *β-actin* to normalize the Cq values of target genes because its expression is stable in *H. diversicolor* and can be used as a housekeeping gene (Li et al., 2012).

### *In situ* hybridization

DNA probes for detecting *HcCNA* and *HcCNB* transcripts were prepared by PCR amplification using a PCR DIG labeling kit (Roche, Mannheim, Germany) with the specific primers shown in Table 1. The amplification program was set as follows: 94 °C for 5 min followed by 35 cycles of 94 °C for 30 s, 52 °C for 30 s, and 72 °C for 30 s and a final extension at 72 °C for 10 min. The PCR products were purified using the FavorPrep GEL/PCR Purification Kit (Favorgen, Ping-Tung, Taiwan) and eluted with DEPC-treated water.

Tissue samples (mantle and gill) were dissected from adult healthy *H. diversicolor* and immediately fixed in 4% paraformaldehyde in PBS at 4 °C overnight. They were washed and dehydrated with ethanol and embedded in paraffin blocks. Five-micron thick sections were prepared, deparaffinized, rehydrated and finally washed with TNE buffer. The samples were treated with 20 µg/ml proteinase K (37 °C, 10 min), fixed with chilled 4% paraformaldehyde, washed in with 0.4% PBST for 5 min, and distilled water before incubating in prehybridization buffer (4 × SSC containing 50% deionized formamide) at 37 °C for 2 h. Thereafter they were incubated in hybridization buffer (4 × SSC, 50% deionized formamide, 50 × Denhard’s solution, 50% W/V Dextran sulfate, 10 mg/ml salmon sperm DNA) containing cDNA probes specific for each gene at 42 °C overnight in a moist chamber. Negative controls, i.e., sections incubated in hybridization buffer without the probe, were also performed. The slides were then washed with 2 × SSC, 1 × SSC, 0.5 × SSC, and 1 × buffer I (1 M Tris–HCl, 1.5 M NaCl). To visualize hybridizing probe, the slides were blocked with 4% BSA and 5% skim milk in buffer I and further incubated with anti-DIG antibody conjugated with alkaline phosphatase. Colorimetric reaction was performed with nitro blue tetrazolium salt and bromo-4-chloro-3-indolyl phosphate in the dark then stopped with TE buffer (100 mM TRIS-HCl, 10mM EDTA). The slides were mounted and viewed under a light microscope without counterstaining.

### Bacterial challenge and sample preparation

To investigate the expression patterns of *HcCNA* and *HcCNB* in response to bacterial challenge, thirty-six abalones were randomly divided into two groups; the bacterial challenge and saline control groups. *V. parahaemolyticus*, XN89, previously isolated from diseased shrimp (Phiwsaiya et al., 2017) was recovered from our −80 °C frozen storage by streaking onto tryptic soy agar (TSA) supplemented with 1.5% NaCl followed by incubation at 30 °C overnight. Bacterial culture was then prepared by inoculating a single colony in 5 ml of tryptic soy broth (TSB) with 1.5% NaCl for 4 h at 30 °C with shaking. The bacterial cell pellet was subsequently collected by centrifugation (3,000 × g, 10 min, 4 °C) and suspended in sterile saline solution (0.85% NaCl). Cells were adjusted to OD_600_ of 0.6 (∼10^8^ cfu/ml as enumerated by the plate count method). This inoculum was appropriately diluted in 0.85% NaCl to prepare for experimental infection. Abalones were injected muscularly via the foot with 25 µl of 2 × 10^6^ cfu/ml *V. parahaemolyticus* (equivalent to 5 × 10^4^ cfu/specimen). For the saline control group, abalones were injected with the same volume (25 µl) of saline. The abalones were returned to their tanks after injection and three abalones from each group were processed for tissue collection at the time intervals of 3, 6, 12, 24, 36, and 48 h post-injection (p.i.). Hemocytes were collected as described above. Mantle, gill, hepatopancreas, and foot were separately collected from each abalone and immediately stored in RNAlater for RNA isolation.

### Quantitative analysis of *HcCNA* and *HcCNB* genes in abalone after bacterial challenge

The expression profiles of *HcCNA* and *HcCNB* after bacterial challenge in various tissues were investigated using real-time PCR. Total RNA from the sampled tissues was isolated and an equal amount of total RNA was converted into cDNA to use as the template in real-time PCR amplification. *HcCNA* and *HcCNB* were amplified by using the primer sequences shown in Table 1. The real-time PCR process was described above and the relative expressions in different tissues were calculated by the Livak (2^−ΔΔ*Cq*^) method.

### Statistical analysis

Data were expressed as mean ± standard deviation. Multiple group comparison was analyzed via ANOVA followed by Duncan’s multiple range test using IBM SPSS Statistics Processor (IBM, Armonk, NY). *P*-value (*p*) < 0.05 was considered statistically significant.

## Results

### Sequencing and phylogenetic analysis of *HcCNA* and *HcCNB*

**Figure 1:**
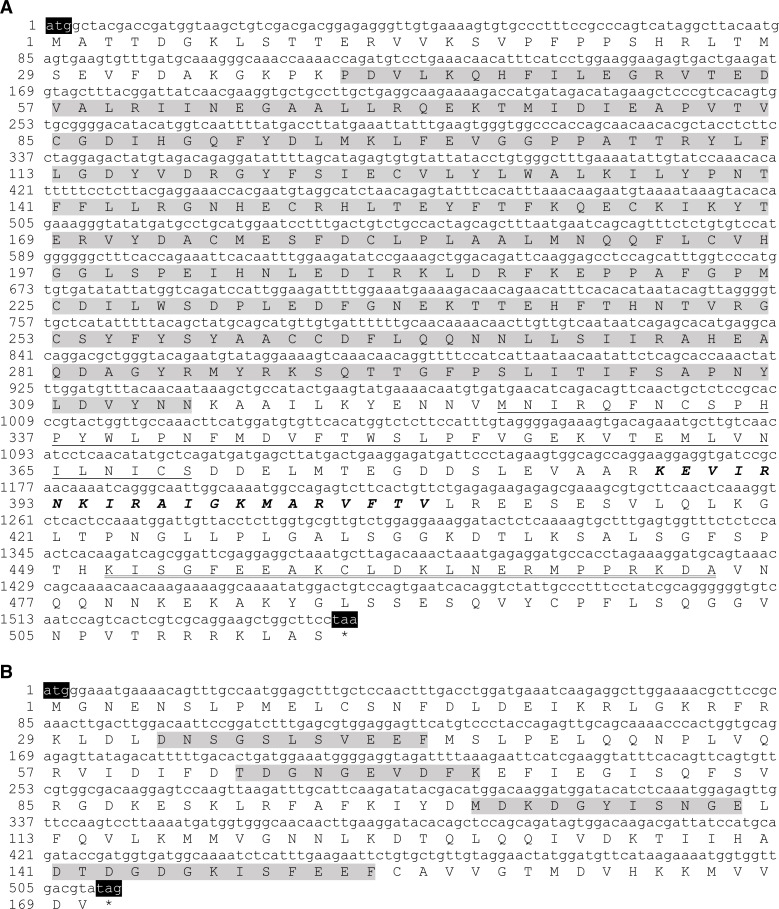
Sequences of open reading frames and their deduced amino acid sequences for the two subunits of *H. diversicolor* calcineurin: *HcCNA* (A) and *HcCNB* (B). Note the four signature domains of *HcCNA* consisting of a catalytic domain (shaded), a CNB binding domain (single underlined), a CaM binding domain (bold and italicized), and an autoinhibitory domain (double underlined). The four EF-hand or calcium-binding domains 1 to 4 of *HcCNB* are shaded.

**Figure 2:**
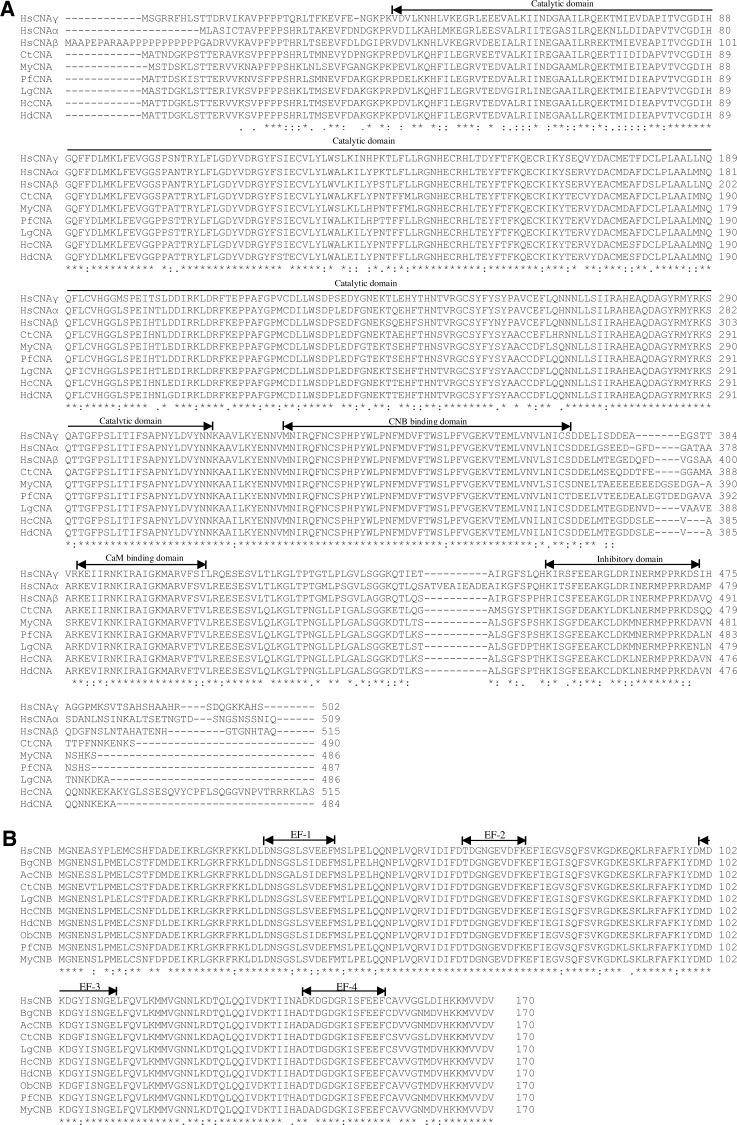
A multiple sequence alignment of *HcCNA*. (A) and *HcCNB* (B) with those of other species using Clustal Omega software. GenBank accession numbers for the amino acid sequences of CNA are as follows: HsCNA *γ*, CNA *γ* isoform (*Homo sapiens*) (NP_001230904.1); HsCNA *α*, CNA *α* isoform (*Homo sapiens*) (XP_016863854.1); HsCNA *β*, CNA *β* isoform (*Homo sapiens*) (NP_001135826.1); CtCNA, CNA (*Capitella teleta*) (ELU02082); MyCNA, CNA (*Mizuhopecten yessoensis*) (XP_021350612.1); PfCNA, CNA (*Pinctada fucata*) (ACI96106); LgCNA, CNA (*Lottia gigantean*) (XP_009062091); HdCNA, CNA (*Haliotis discus discus*) (ABO26624). GenBank accession numbers for the amino acid sequences of CNB are as follows: HdCNB, CNB (*H. discus discus*) (ABO26623); PfCNB, CNB (*Pinctada fucata*) (ACI96107); LgCNB, CNB (*Lottia gigantean*) (XP_009055806); ObCNB, CNB (*Octopus bimaculoides*) (XP_014783704.1); MyCNB, CNB (*Mizuhopecten yessoensis*) (XP_021367340.1); BgCNB, CNB (*Biomphalaria glabrata*) (XP_013072348); AcCNB, CNB (*Aplysia californica*) (XP_005089145); CtCNB,CNB (*Capitella teleta*) (ELT98040); HsCNB, CNB (*Homo sapiens*) (NP_000936). *, identity; :, close similarity; ., more distant similarity.

cDNAs encoding the *HcCNA* protein were obtained by RT-PCR from the mantle tissue of *H. diversicolor*. The *HcCNA* open reading frame (ORF) is 1,548 nucleotides long (Fig. 1A), and is predicted to encode 515 amino acid (aa) protein with a calculated molecular mass of 58.51 kDa and a theoretical isoelectric point (pI) of 6.48. The cDNA sequence of *HcCNA* was submitted to GenBank with the accession no. MN635462. The predicted protein contained all CNA signature domains including a catalytic domain (positioned at 40-314 aa, grey highlight), a CNB binding domain (326-370 aa, single underlined), a calmodulin (CaM) binding domain (388-407 aa, bold and italicized), and an autoinhibitory domain (451-474 aa, double underlined). The amino acid sequence was further compared with CNA sequences of other species (Fig. 2A), and showed high sequence similarity to *Haliotis discus discus* CNA (GenBank accession no. ABO26624), *Lottia gigantea* CNA (XP_009062091), *Pinctada fucata* CNA (ACI96106), and *Mizuhopecten yessoensis* CNA (XP_021350612.1), with 90 to 99 percent identities. When compared to the amino acids sequences of three isoforms of human CNA (*HsCNAα*, *HsCNAβ*, *HsCNAγ*), *HcCNA* showed 80%, 79%, and 75% identities, respectively. Like other mollusks, the HcCNA sequence lacked the polyproline region found in mammalian CNA*β*.

A 513bp ORF cDNA of *HcCNB* was amplified from the mantle tissue of *H. diversicolor*. The cDNA sequence of *HcCNB* was submitted to GenBank with the accession no. MN635463. Figure 1B shows the deduced 170 aa HcCNB protein which consists of 4 conserved EF-hand type calcium binding motifs including EF-1 (33-43 aa), EF-2 (64-73 aa), EF-3 (101-111 aa), and EF-4 (141-153 aa), with a calculated molecular weight of 19.33 kDa and an isoelectric point of 4.57. HcCNB was used in homology searches against the GenBank protein database (Fig. 2B). The deduced amino acid sequence of HcCNB shared the highest similarity to *H. discus discus* CNB (GenBank accession no. ABO26623) with 100% identity. It also shares high similarity to *Pinctada fucata* CNB (ACI96107), *Lottia gigantea* CNB (XP_009055806), *Octopus bimaculoides* CNB (XP_014783704.1), *Mizuhopecten yessoensis* CNB (XP_021367340.1), *Biomphalaria glabrata* CNB (XP_013072348), *Aplysia californica* CNB (XP_005089145), *Capitella teleta* CNB (ELT98040), and *Homo sapiens* CNB (NP_000936) with 90%–96% identities.

**Figure 3:**
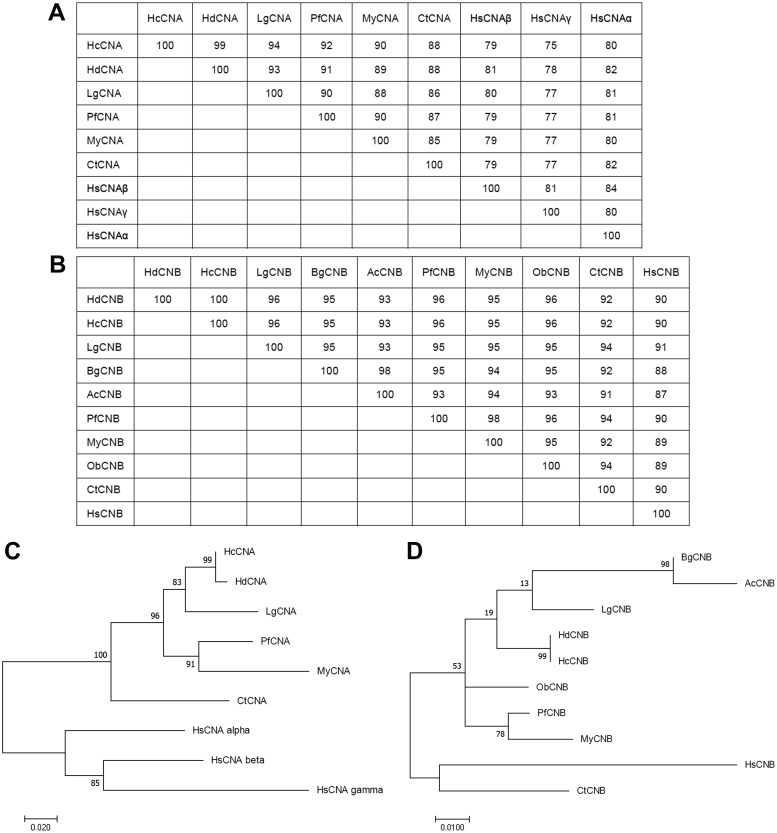
Phylogenetic analysis of HcCNA (A and C) and HcCNB (B and D) with other species. The trees were produced using the Mega7 software with the maximum-likelihood method and were based on the multiple sequence alignment shown in Fig. 2. Percent bootstrap values are indicated on the branches. Scale bars indicate the branch length for the stipulated number of amino acid substitutions. The tables above the trees indicated their sequence similarities.

Phylogenetic analysis of the HcCNA and HcCNB protein sequences with CN sequences from other species was performed using a maximum-likelihood method (Figs. 3A to 3D). The numbers at the branch nodes showed relatively high percent bootstrap confidence values overall, with both HcCNA and HcCNB grouping with the corresponding *H. discus discus* CN protein subunits with high support (99%).

### Tissue distributions of *HcCNA* and *HcCNB* in *H. diversicolor*

**Figure 4:**
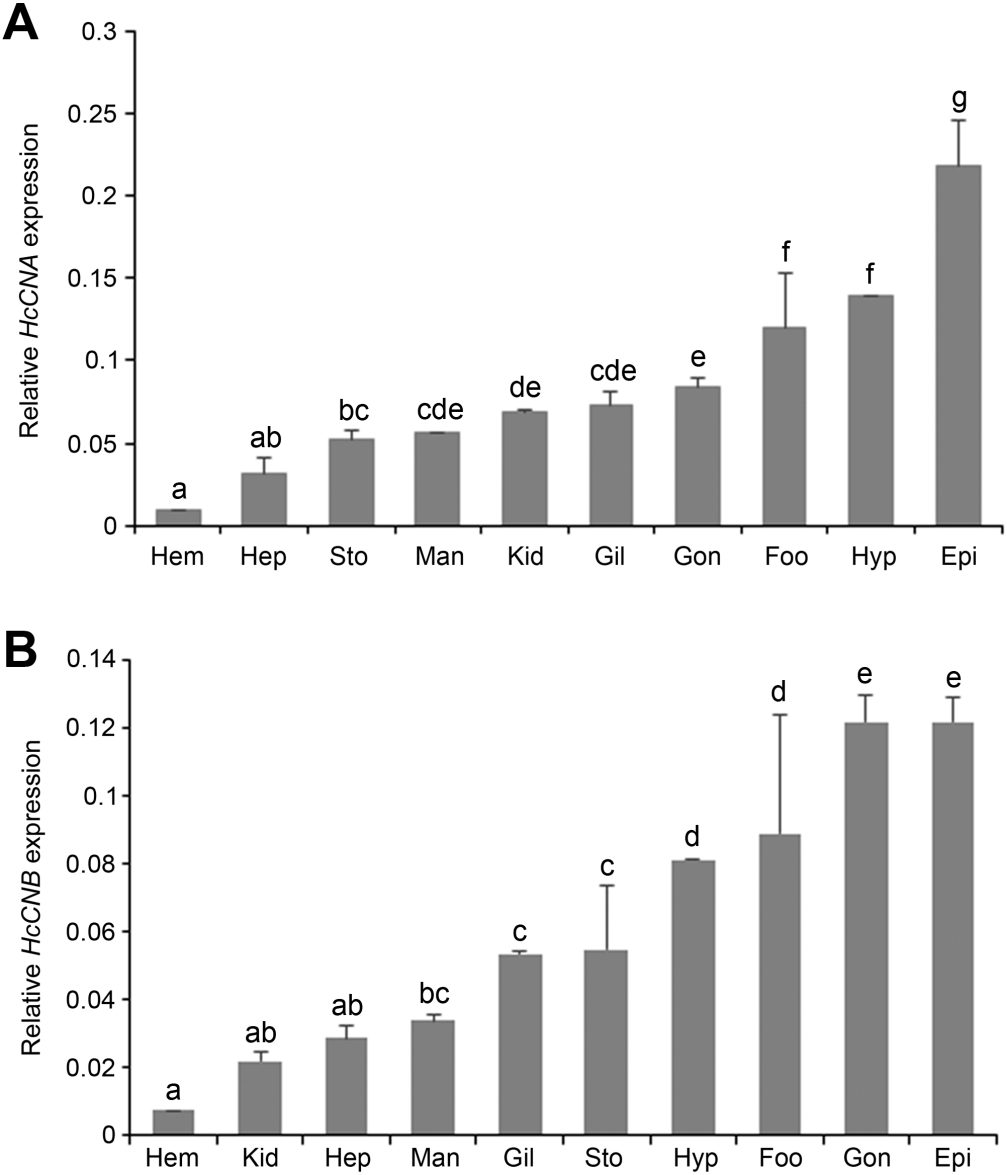
Tissue distributions of *HcCNA*. (A) and *HcCNB* (B) of *H. diversicolor* analyzed by real-time PCR. The relative expression was calculated by the Livak method (2^ΔΔ*Cq*^) using *β*- *actin* as a reference gene. Data was presented as mean relative expression of triplicate real-time reactions. Hem, hemocyte; Gil, gill; Hyp, hypobrachial gland; Man, mantle; Epi, epipodium; Gon, gonad; Sto, stomach; Foo, foot; Hep, hepatopancreas; Kid, kidney. Lowercase letters indicated significant differences at *p* < 0.05.

Quantitative real-time PCR was performed to determine the expression of *HcCNA* and *HcCNB* transcripts in different abalone tissues: mantle, gill, epipodium, hepatopancreas, gonad, stomach, foot, kidney, hypobranchial gland, and hemocytes. The mRNA expression of each tissue was normalized to that of *β*-*actin* as a reference gene. Expression results showed that both *HcCNA* (Fig. 4A) and *HcCNB* (Fig. 4B) were ubiquitously expressed in all examined tissues. The highest expression levels of *HcCNA* and *HcCNB* genes were found in epipodium and the lowest expression levels were found in hemocytes when compared to all the analyzed tissues.

**Figure 5:**
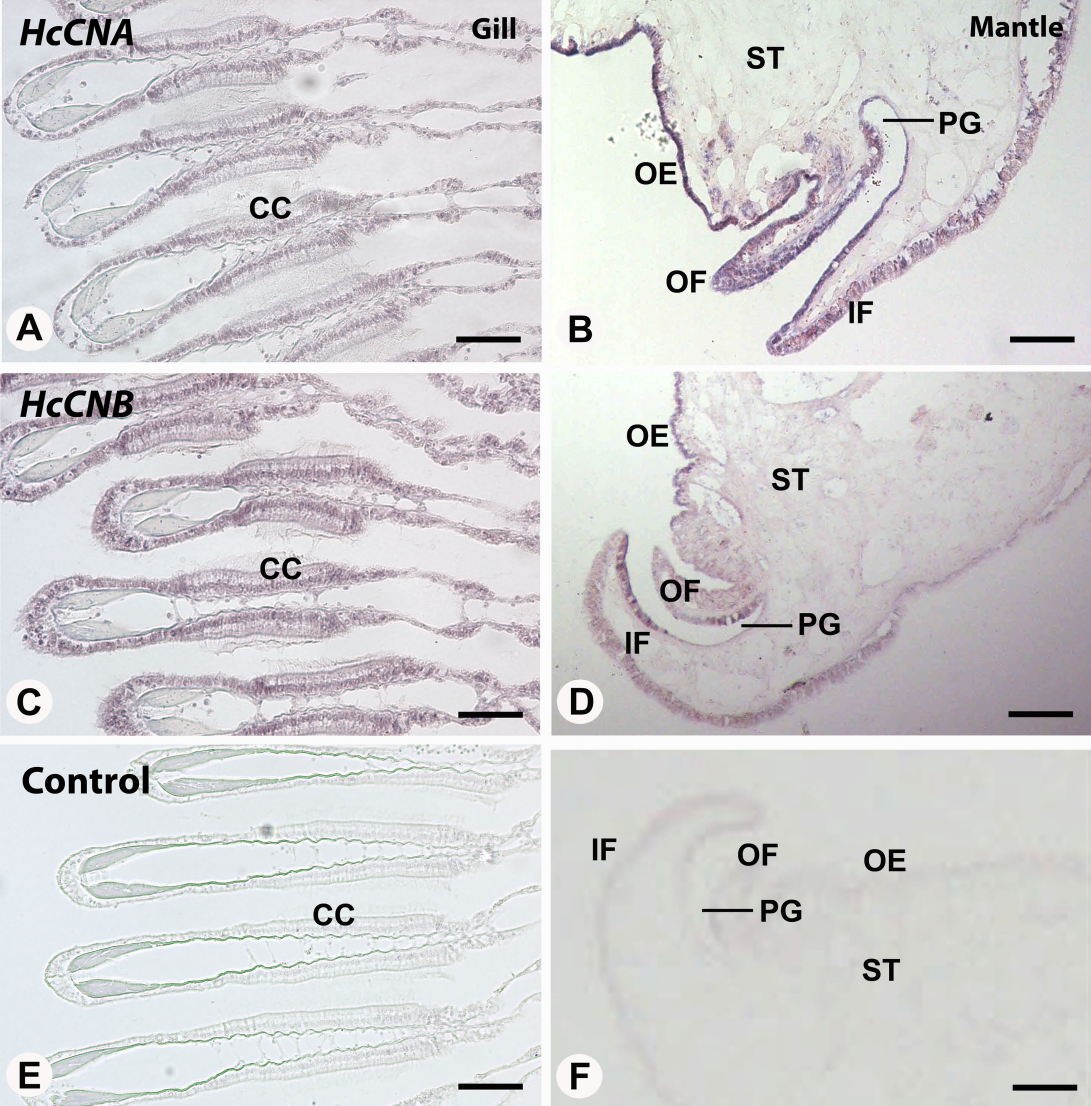
*In situ* hybridization of *HcCNA* (A and B) and *HcCNB* (C and D) in the mantle and gill of *H. diversicolor*. Note the intense staining in the ciliated columnar epithelial cells (CC) in gill and along the outer epithelium (OE) of the mantle tissue. (E) and (F) Represent negative controls. OF, outer fold; IF, inner fold; PG, periostracal groove; ST, stromal tissue. Bars = 100 µm.

We also performed *in situ* hybridization of both *HcCNA* and *HcCNB* genes in the two exposed immune related tissues, gill and mantle. In the mantle, hybridization signal of *HcCNA* was observed in the entire length of the mantle epithelial cells (Fig. 5B). An intense reactivity of *HcCNA* probe was detected in the outer epithelial cells of the mantle pallial. Likewise, a strong hybridization signal of *HcCNB* was observed in the outer epithelial cells of the mantle pallial, but it did not span the whole length of epithelium (Fig. 5D). In the gill, reactivity of both *HcCNA* and *HcCNB* probes (Figs. 5A and 5C) was observed in a single cell layer of the entire gill epithelium. The signals were detected intensely in the nuclei as well as in the cytoplasm of the epithelial cells where the mRNA of both genes are localized. Negative controls (Figs. 5E and 5F) did not show any hybridization signal.

### *HcCNA* and *HcCNB* gene expression patterns in response to bacterial challenge

**Figure 6:**
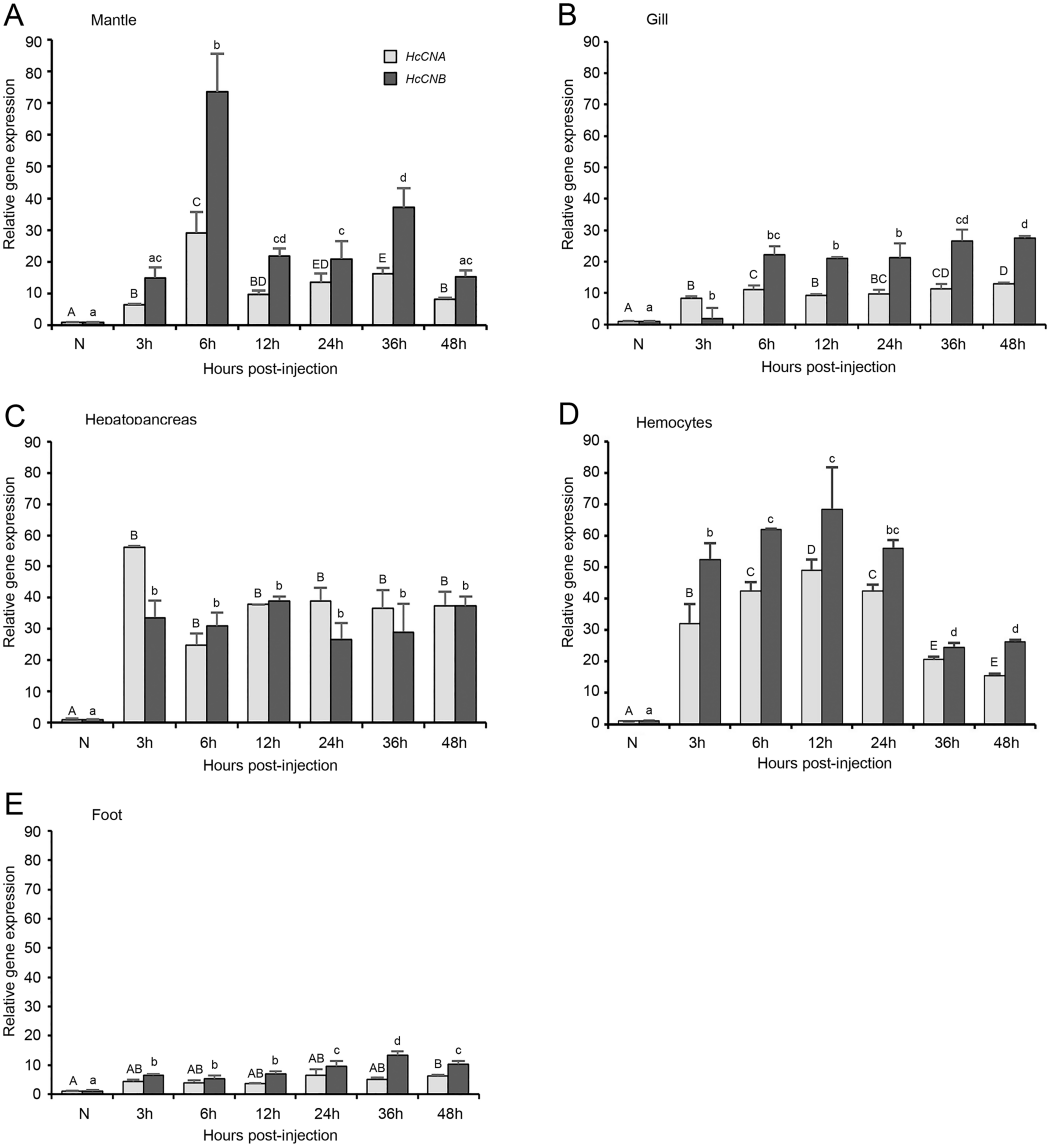
*HcCNA* and *HcCNB* gene expression patterns in response to *V. parahaemolyticus* challenge. The expression patterns of *HcCNA* and *HcCNB* were detected by quantitative real-time PCR in the mantle, gill, hepatopancreas, hemocytes, and foot (A, B, C, D, and E). The data was shown expression levels relative to the baseline. *β*-*actin* was used as an internal reference gene. Data denoted with difference uppercase and lowercase letters indicated significant difference at *p* < 0.05. Solid bars = *HcCNA* gene; sparse bars = *HcCNB* gene.

We performed bacterial challenge test by injecting 5 × 10^4^ cfu into an individual abalone and measured the expression of *HcCNA* and *HcCNB* genes at different time intervals. It should be noted that the injection dose used in this study was considered a sub-lethal dose, namely, there was no abalone mortality observed in both bacterial exposure and control groups during the 48 h challenge test. However, at 48 h. p.i. the infected abalone exhibited loose attachment to the rearing tank due to foot muscle weakness. Generally, both *HcCNA* and *HcCNB* genes were highly up-regulated in the immune-related tissues including mantle, gill, hepatopancreas, and hemocytes (Figs. 6A–6D), but at a low to moderate level in the non-immune related tissues (foot (Fig. 6E)), upon the bacterial infection. In the mantle of *V. parahaemolyticus* challenged animals, *HcCNA* mRNA was slightly up-regulated at 3 h p.i. and peaked at 6 h p.i. followed by its gradual decline at 12 h p.i. and in the longer period up to 48 h p.i. Interestingly, *HcCNB* was drastically up-regulated at 3 h p.i. which was twofold higher than that of *HcCNA*. *HcCNB* expression levels peaked at 6 h p.i. (2.5 fold higher than *HcCNA*), and rapidly ceased at 12, 24 and 48 h p.i. although a slight increase was noted at 36 h p.i. Similar trends were also observed in gill tissues, namely, both genes were slightly up-regulated at 3 h p.i. and highly up-regulated at 6 h p.i., but the expression was maintained at high levels for at least 48 h p.i. The hepatopancreas showed rapid increase in expression of both *HcCNA* and *HcCNB* as early as 3 h p.i., where the increased level went up to >10 fold. The expression levels were gradual decreased in the later time intervals up to 48 h p.i. Compared to the other tissues studied, the levels of *HcCNB* in hepatopancreas was usually lower than that of *HcCNA,* a pattern that was not common in other tissues examined. In hemocytes, the expression of both *HcCNA* and *HcCNB* were rapidly up-regulated at 3 h p.i. and highly up-regulated at 6 and 12 h p.i. followed by their gradual decrease at 24, 36, and 48 h p.i. In the foot, *HcCNA* was slightly up-regulated at 3 h p.i. and maintained at a high expression level for at least 48 h p.i., however, the expression levels in this tissue was considerably lower than that observed elsewhere.

## Discussion

In this study, we have characterized both *HcCNA* and *HcCNB* in *H. diversicolor.* These genes show characteristics similar to other studied CN subunits. *HcCNA* possesses the typical four conserved domains while *HcCNB* is composed of EF hand motifs (Rusnak & Mertz, 2000). However, we only found one unique cDNA sequence for *HcCNA* in the sequenced cDNA library compared to the existing 3 isoforms (CNA*α*, *β* and *γ*) of catalytic subunits found in human (*HsCNA*) (Muramatsu & Kincaid, 1992). Previous analyses have shown that mollusk CNA isoforms, such as CNA isolated from *M. yesssoensis* (*MyCNA*; Uryu et al., 2000) and *Pinctada fucata* (*PfCNA*; Li et al., 2009), showed high similarity with the mammalian brain-type isoform (CNA*α*) rather than the testis-specific isoform (CNA*γ*). Furthermore, all analyzed mollusk CNA sequences lack the proline-rich domain at the *N*-terminal sequence that is found in *HsCNAβ* (Guerini & Klee, 1989). Unlike CNA, which has undergone the aforementioned evolutionary changes, CNB possesses high degree of conserved amino acid sequence identities from yeast to mammals, including the *HcCNB* characterized in this study (Fig. 1). In addition, the phylogenetic analysis in Fig. 2 clearly confirmed that both *HcCNA* and *HcCNB* are closely related to CN subunits in other mollusk species.

The distribution of CN transcripts is widely variable among mammalian tissues with the highest levels found in brain (Klee, Draetta & Hubbard, 1988), whereas they are predominantly reported in immune related tissues in many invertebrates. A considerable level of CNA expression is detected in the hemocytes of the pearl oyster, *P. fucata* (Li et al., 2010a)*,* and the Chinese mitten crab, *E. sinensis* (Li et al., 2015). In our study, the *HcCNA* and *HcCNB* transcripts are ubiquitously expressed in all examined tissues, including hemocytes. As derived from the study in *P. fucata*, CN mediates the immune response of hemocytes via activating NF- *κ*B signaling pathway (Li et al., 2010b). The activated CN, possibly synergized with PKC, stimulated IKK, through which I*κ*B*α* was phosphorylated, ubiquitinated and degraded by the proteasome complex to release it from NF- *κ*B which consequently bind to the promotors of IL-2 and iNOS. Both IL-2 and iNOS are important defenders in the innate immune system of pearl oyster to fight against the invading pathogens (Li et al., 2010b).

Infection of *V. parahaemolyticus* in many marine animals such as crabs, shrimp, oysters, and abalone has resulted in various disease symptoms. The most serious case of *V. parahaemolyticus* infection is found in abalone, in which the pathogen causes withering syndrome and leads to lethargy, retracted visceral tissues, and atrophy of the foot muscle (Liu et al., 2000). The involvement of a number of genes in the molecular response to *V. parahaemolyticus* infection in the abalone has been shown experimentally by assessing gene expression during bacterial challenge tests. Up-regulated genes include Hdh-cSP (Hu et al., 2018), cathepsin Z (Godahewa et al., 2017), HdiQM (Wei-Dong et al., 2016), cathepsin L (Shen et al., 2015), IGFBP7 (Li et al., 2012), caspase 8 and 10 (Huang et al., 2010), and Ab-CaReg1 (Nikapitiya et al., 2010) in response to the bacterial challenge. In addition, the up-regulation of these genes was detected in multiple tissues including the gill, hepatopancreas, mantle, and muscle, supporting the notion that multiple tissues are involved in invertebrate immune responses. In this study, we found that both *HcCNA* and *HcCNB* rapidly increased in the same immune related tissues (Fig. 6) as those in *E. sinensis,* where both *EsCN-A* and *EsCN-B* are up-regulated in hepatopancreas, gill, and hemocytes (Li et al., 2015). Upon infection of *Vibrio* through 3 different routes (oral feeding, immersion and injection), its spreading throughout the body has been commonly modulated via hemocytes and hemolymph (Alday-Sanz, Roque & Turnbull, 2002). This infection mechanism should therefore explain the accumulation of *Vibrio* and enhancement of *HcCNA* and *HcCNB* (and may be other immune-related genes) in a broad array of tissues (Fig. 6). Apart from hemocytes, the involvement of gill and hepatopancreas as immune responsive organs at the time of microbe infections in marine invertebrates has been gradually accumulated (Ji, Yao & Wang, 2009; Röszer, 2014; Li et al., 2013). In mollusks and crustaceans, both hepatopancreas and gill are known to endogenously synthesize innate immune-related molecules such as C-type lectin and anti-microbial proteins against the bacterial challenge (Röszer, 2014). In addition, knocking down of MjGCTL (gill C-type lectin) in *M. japonicas* has resulted in an impairment of bacterial agglutination ability and ceased levels of crustin and penaeidin (anti-microbial peptides) in gill tissues (Alenton et al., 2019). More interestingly, independent functions of CNB in many biological processes have been well recognized (Wu et al., 2016; Yang et al., 2016; Zhang et al., 2017), indicating that CNA and CNB may be independently regulated. This may explain the differences in expression patterns observed for *HcCNA* and *HcCNB* in different tissues (Fig. 4), and in response to bacterial challenge (Fig. 6).

## Conclusion

Both calcineurin genes, *HcCNA* and *HcCNB*, were characterized in the colored abalone, *H. diversicolor*. These genes were constitutively distributed in the selected tissues of abalone. The expression levels of both calcineurin genes were significantly enhanced in hemocytes, hepatopancreas, gill and mantle upon *Vibrio* infection. This study provided fundamental knowledge about innate immune response in mollusk against microbes and open up a strategy of triggering innate immune response to fight against bacterial invasion.

##  Supplemental Information

10.7717/peerj.8868/supp-1 Figure S1 Nucleotide sequences of *HcCNA* and *HcCNB* that are submitted and processed by GenBank

10.7717/peerj.8868/supp-2 Supplemental Information 2 Spreadsheet of qPCR data of Figure 4

10.7717/peerj.8868/supp-3 Supplemental Information 3 Spreadsheet of qPCR data of Figure 6

## Additional Information and Declarations

### DNA Deposition

The following information was supplied regarding the deposition of DNA sequences: The raw data is available at GenBank: MN635462, MN635463.

### Data Availability

The following information was supplied regarding data availability: The Nucleotide sequences of HcCNA and HcCNB are available at GenBank: BankIt2278227 Seq1 MN635462, BankIt2278227 Seq2 MN635463. The sequences are also available in Fig. S1. The raw data set of qPCR data for Figure 4 (tissue distribution of *HcCNA* and *HcCNB*) and Figure 6 (bacterial challenge) are available in the Supplemental Files.
