# Supplementary material for: Characterization of calcineurin A and B genes in the abalone, Haliotis diversicolor, and their immune response role during bacterial infection
Source: PeerJ. 2020 Apr 9;8:e8868. doi: 10.7717/peerj.8868 (PMC7151749; doi:10.7717/peerj.8868)
Supplement: Figure S1 [file peerj-08-8868-s001.pdf]

Sequence of open reading frame of *HcCNA* from *Haliotis diversicolor* (GenBank accession number MN635462)

ATGGCTACGACCGATGGTAAGCTGTCGACGACGGAGAGGGTTGTGAAAAGTGTGCCCTTTCC
GCCCAGTCATAGGCTTACAATGAGTGAAGTGTTTGATGCAAAGGGCAAACCAAAACCAGATG
TCCTGAAACAACATTTCATCCTGGAAGGAAGAGTGACTGAAGATGTAGCTTTACGGATTATC
AACGAAGGTGCTGCCTTGCTGAGGCAAGAAAAGACCATGATAGACATAGAAGCTCCCGTCAC
AGTGTGCGGGGACATACATGGTCAATTTTATGACCTTATGAAATTATTTGAAGTGGGTGGCC
CACCAGCAACAACACGCTACCTCTTCCTAGGAGACTATGTAGACAGAGGATATTTTAGCATA
GAGTGTGTATTATACCTGTGGGCTTTGAAAATATTGTATCCAAACACATTTTTCCTCTTACG
AGGAAACCACGAATGTAGGCATCTAACAGAGTATTTCACATTTAAACAAGAATGTAAAATAA
AGTACACAGAAAGGGTATATGATGCCTGCATGGAATCCTTTGACTGTCTGCCACTAGCAGCT
TTAATGAATCAGCAGTTTCTCTGTGTCCATGGGGGGCTTTCACCAGAAATTCACAATTTGGA
AGATATCCGAAAGCTGGACAGATTCAAGGAGCCTCCAGCATTTGGTCCCATGTGTGATATAT
TATGGTCAGATCCATTGGAAGATTTTGGAAATGAAAAGACAACAGAACATTTCACACATAAT
ACAGTTAGGGGTTGCTCATATTTTTACAGCTATGCAGCATGTTGTGATTTTTTGCAACAAAA
CAACTTGTTGTCAATAATCAGAGCACATGAGGCACAGGACGCTGGGTACAGAATGTATAGGA
AAAGTCAAACAACAGGTTTTCCATCATTAATAACAATATTCTCAGCACCAAACTATTTGGAT
GTTTACAACAATAAAGCTGCCATACTGAAGTATGAAAACAATGTGATGAACATCAGACAGTT
CAACTGCTCTCCGCACCCGTACTGGTTGCCAAACTTCATGGATGTGTTCACATGGTCTCTTC
CATTTGTAGGGGAGAAAGTGACAGAAATGCTTGTCAACATCCTCAACATATGCTCAGATGAT
GAGCTTATGACTGAAGGAGATGATTCCCTAGAAGTGGCAGCCAGGAAGGAGGTGATCCGCAA
CAAAATCAGGGCAATTGGCAAAATGGCCAGAGTCTTCACTGTTCTGAGAGAAGAGAGCGAAA
GCGTGCTTCAACTCAAAGGTCTCACTCCAAATGGATTGTTACCTCTTGGTGCGTTGTCTGGA
GGAAAGGATACTCTCAAAAGTGCTTTGAGTGGTTTCTCTCCAACTCACAAGATCAGCGGATT
CGAGGAGGCTAAATGCTTAGACAAACTAAATGAGAGGATGCCACCTAGAAAGGATGCAGTAA
ACCAGCAAAACAACAAAGAAAAGGCAAAATATGGACTGTCCAGTGAATCACAGGTCTATTGC
CCTTTCCTATCGCAGGGGGGTGTCAATCCAGTCACTCGTCGCAGGAAGCTGGCTTCCTAA

Sequence of open reading frame of *HcCNB* from *Haliotis diversicolor* (GenBank accession number MN635463)

ATGGGAAATGAAAACAGTTTGCCAATGGAGCTTTGCTCCAACTTTGACCTGGATGAAATCAA
GAGGCTTGGAAAACGCTTCCGCAAACTTGACTTGGACAATTCCGGATCTTTGAGCGTGGAGG
AGTTCATGTCCCTACCAGAGTTGCAGCAAAACCCACTGGTGCAGAGAGTTATAGACATTTTT
GACACTGATGGAAATGGGGAGGTAGATTTTAAAGAATTCATCGAAGGTATTTCACAGTTCAG
TGTTCGTGGCGACAAGGAGTCCAAGTTAAGATTTGCATTCAAGATATACGACATGGACAAGG
ATGGATACATCTCAAATGGAGAGTTGTTCCAAGTCCTTAAAATGATGGTGGGCAACAACTTG
AAGGATACACAGCTCCAGCAGATAGTGGACAAGACGATTATCCATGCAGATACCGATGGTGA
TGGCAAAATCTCATTTGAAGAATTCTGTGCTGTTGTAGGAACTATGGATGTTCATAAGAAAA
TGGTGGTTGACGTATAG
